# ‘Who cares if you're poz right now?’: Barebackers, HIV and COVID‐19

**DOI:** 10.1111/1467-9566.13369

**Published:** 2021-09-09

**Authors:** Jaime Garcia‐Iglesias, Chase Ledin

**Affiliations:** ^1^ Centre for Biomedicine, Self and Society University of Edinburgh Edinburgh UK

**Keywords:** bareback, COVID‐19, HIV/AIDS, prevention, responsibility

## Abstract

The global COVID‐19 pandemic poses new challenges for communities built around certain sexual practices, and some of which have responded by using their previous experiences of HIV. In this article, we undertake an online ethnography of a popular Anglo‐American barebackers' forum to understand how HIV and COVID‐19 converge and how these men negotiate COVID‐19 risk by adapting previous sexual and disease prevention strategies. Barebackers, aka gay men who eroticise condomless anal intercourse, provide a relevant group to consider given their longstanding negotiation of HIV. We explore processes of responsibility, risk management and pleasure during the COVID‐19 pandemic. We suggest that their experiences of both the AIDS crisis and the current context of HIV frame their decisions around COVID‐19. We focus on how responsibility and desire shape discussions of bathhouses and the survival of barebackers' sexual practices during and after COVID‐19.

**Abbreviations**
AIDS: acquired immune deficiency syndrome
ART: antiretrovirals
CFR: case fatality rate
COVID-19: coronavirus disease 2019
HIV: human immunodeficiency virus
PEP: post-exposure prophylaxis
PrEP: pre-exposure prophylaxis
U=U: undetectable equals untransmittable

## INTRODUCTION

COVID‐19^1^ poses a unique problem for many sexual practices, including those already disproportionately impacted by HIV. As societies struggle to provide effective risk‐management policies, some groups have recognised similarities between HIV and COVID‐19 and have discussed them in light of their sexual and social practices (Osmundson, 2020; Picardi, 2020; Preciado, 2020). In this article, we focus on how a specific sexual subculture—barebackers (i.e. gay men who eroticise condomless anal intercourse)—has responded to COVID‐19 using varied experiences and memories of HIV and AIDS.^2^ We have chosen to focus on barebackers because of the abundance of literature discussing their practices in terms of viral risk (or lack of risk). Focusing on how barebackers negotiate COVID‐19 is enlightening because this group has been historically affected by HIV transmission, and much of its practices revolve around the risk of infection. In exploring how HIV and COVID‐19 converge in their discourses, we address two important questions: First, how do barebackers engage with their experiences of the AIDS crisis and HIV to make sense of COVID‐19? Second, how do they negotiate sexual practices and prevention in a time of COVID‐19 transmission?

To answer these questions, this article draws on an online ethnography of a popular Anglo‐American barebacking forum conducted in early April 2020. We analyse, first, how barebackers' engagement with COVID‐19 and its prevention in relation to both their experiences of past and present HIV. We then argue that, in relation to these experiences, barebackers engage in a complex negotiation of pleasure, desire and responsibility both within their own subcultural groups and at broader societal levels. Throughout this article, we use a qualitative perspective to expand ongoing discussions about the relationships between HIV and COVID‐19, crystallising debates about the character of barebacking and providing a discussion of how these men understand, negotiate and seek to prevent COVID‐19. This article does not only provide a snapshot of the COVID‐19 crisis as it affects a particular sexual subculture, but also raises questions about how prevention discourses, sexual health and risk reduction may be understood as the HIV and COVID‐19 pandemics continue to intertwine in unique and unforeseeable ways.

We begin by discussing the ways in which HIV and COVID‐19 have been compared, examining affective similarities and highlighting important differences. As part of our analytic framework, we provide a definition and discussion of barebackers and their relationship to HIV. We discuss the methodological benefits of an online ethnography while remaining attentive to its limitations. Drawing from our ethnographic dataset, we narrate key theoretical developments structured within discussions of barebackers' practices and COVID‐19 pandemic. First, we explore how barebackers deploy their experiences of HIV to negotiate and make sense of the COVID‐19 pandemic. Second, we explore how these men engage in discussions of responsibility and desire. Then, we develop this by exploring how responsibility and desire frame discussions of bathhouses and the survival of barebackers' sexual practices. We conclude by suggesting some of the implications of these findings for our understandings of barebacking and risk management.

## CONTEXT

### HIV and COVID‐19: viral comparisons

Reflecting on novelist Andrew Holleran's work, poet and author Garth Greenwell (2020) has written that the COVID‐19 pandemic reminds him of the early AIDS crisis^3^:

“I have felt an eerie affective familiarity during the past several weeks, in my anger at governmental failings and in the uncertainty I've felt in the face of contradictory information about who is at risk and to what degree, about where risk lies and how we should protect ourselves, about how many are likely to die.”

Greenwell describes the ‘affective familiarity’ between the AIDS crisis and COVID‐19 as anchored in feelings of ‘anger’ and ‘uncertainty’ produced by ineffective governments and a lack of clarity about ‘risk’ and ‘protection’. Though this practice of paralleling responses to epidemics is not unique (Snowden, 2019), Greenwell conjures memories of the sexual politics of AIDS to describe how societies like the United States perpetually fail to provide effective national and global health strategies due to the lack of clear information about transmission and protection.

Comparing epidemics often occurs through the entanglement of historical analysis, epidemiology, cultural anthropology and social theory. In a new preface to *Epidemics and Society*, for instance, medical historian Frank Snowden writes: ‘Like all pandemics, COVID‐19 is not an accidental or random event. Epidemics afflict societies through the specific vulnerabilities people have created by their relationships with the environment, other species, and each other’ (2019, ix). Grounded in networks of human and non‐human actors, Snowden suggests several points of historical comparison, including causative pathogens, rates of mortality and morbidity, case fatality rates (CFR), symptoms, population demographics, mode(s) of transmission and disease duration (83–87). Like Snowden, other scholars have examined the links between socioeconomic inequalities and CFR (Thrasher, 2020), the transformation of scientific expertise (Engelmann, 2020; Epstein, ), and governance and medical surveillance (Quah, 2007). These scholars call upon previous ways of knowing about and responding to epidemics in order to demonstrate how technoscientific responses adapt and evolve (Clarke et al., 2010). This includes the transformation of public health procedures in order to describe and assess the uncertain social conditions that might result from COVID‐19 pandemic (Osmundson, 2020; Picardi, 2020; Preciado, 2020), and the changing social dynamics that reveal the ‘affective familiarities’ across experiences of illness (Cvetkovich, 2003; Gould, 2009). Building upon the tensions that emerge through these many ways of knowing about epidemics across history, we contribute to this discourse by exploring how barebackers, a subcultural group historically disproportionately affected by HIV, use the experiences of HIV to negotiate prevention strategies for COVID‐19.

Many scholars have carefully detailed the differences between the HIV and COVID‐19 pandemics, their social conditions and the viruses that produce them (Bowleg, 2020; Jones, 2020; Logie & Turan, 2020). Some authors also make important distinctions based on the affected populations. García‐Iglesias and Nagington (2020), for example echo Jeffrey Week's quote that ‘AIDS was the disease of the gays, the prostitutes, the intravenous drug users, and all those who belonged to the already stigmatised groups of society’ (1981 [2012], 381). They suggest that early descriptions of the AIDS pandemic acknowledged the emergence of multiple epidemics which helped to illuminate the uneven distribution of illness (Cheng et al., 2020). In particular, García‐Iglesias and Nagington (2020) suggest that the early images associated with the AIDS crisis (e.g. emaciated young men, parties and sexualised people) were dramatically different from the images of middle‐class, white, heterosexual cruise‐ship passengers portrayed in early COVID‐19 reporting in the United Kingdom and the United States (BBC, 2020). These contrasts are even stronger due to the patent differences in routes of transmission: whereas COVID‐19 is mostly airborne, HIV remains largely transmitted through either sexual intercourse or intravenous drug use, which has historically subjected and continues to subject these bodies to further particularly insidious stigmatisation (Hutchinson & Dhairyawan, 2017).

Scholars has begun to describe and analyse the affective familiarities of these pandemics, noting the *potential* for experiences of the AIDS pandemic and of current HIV to illuminate responses to COVID‐19 (e.g. Day, 2021; Ledin & Weil, 2021).^4^ Their reflections help to provide greater insight into how understandings of COVID‐19 draw upon and reframe memories of AIDS crisis and experiences of HIV. This is a thriving area of work, with special journal issues being advertised, academic guidelines produced, and online seminars held (e.g. Florêncio, 2020; HIV Doula, 2020; Osmundson, 2020; Picardi, 2020; Preciado, 2020)––and from which our paper follows.

### Barebacking

We draw on two social theorists, Tim Dean (2009) and João Florêncio (2020), whose analyses of the transformation of gay‐male sexual cultures since the emergence of combination antiretroviral therapy (ART)^5^ provide a framework for understanding barebacking today. Barebacking is said to have originated in the late 1990s coinciding with the introduction of ART, which allowed those who had access to ART to transition from experiences of fatal disease to chronic illness (Race, 2009). Since then, barebacking has shifted from a taboo practice to a popular social practice among some gay men that is commonly represented in pornography (Morris & Paasonen, 2014). Tim Dean has defined barebacking as a ‘subculture’ of gay men characterised by the eroticising of anal sex without condoms (2009, ix). Assessing the psychoanalytic frameworks that shape these practices, Dean acknowledges that it is difficult to separate barebacking from other practices, such as condomless anal sex in long‐term monogamous relationships and ‘bugchasing’. He demonstrates how specific ‘raw’ desires are assembled through these community practices, articulating not simply the ‘self‐shattering’ rupture of potential HIV infection, but noting the ways in which queer kinship is negotiated through the exchange of fluids. This has led other authors to suggest that barebacking is rather an umbrella term for a ‘full range of relations to unprotected sex’ (Race, 2015b, 24). Recent debates on the subject focus on exploring its demographics, history and current appeal in the age of pre‐exposure prophylaxis (PrEP)^6^ (Race, 2009; Varghese, 2019).

Building on Dean's work, João Florêncio argues that ‘condomless sex has been decoupled from the spectre of AIDS’ resignifying how ‘gay men engag[e] in ‘bareback’ or ‘raw’ sex’ (2020, 12). This decoupling has allowed for reconsideration of sexual practices as perceptions of HIV risk have changed with increasing PrEP use: In fact, the advent of biomedical prevention and treatment tools (such as PrEP, PEP and treatment‐as‐prevention) have made it possible for many to engage in barebacking with no risk of HIV (Garcia Iglesias, 2020a, 2021a). The culture of barebacking, which Florêncio calls ‘pig sex,’ is supported by scientific confidence in ART’s efficacy, including undetectability (U = U)^7^ for HIV‐positive individuals and the use of PrEP for HIV‐negative individuals. As such, it is a culture in which participants engage in condomless sex and explore new forms of care through their use and knowledge of prophylactic technologies. In other words, barebackers have particular knowledge not only of their desires for condomless sex, but also a willingness to include and negotiate prophylactic measures (e.g. ART, PEP^8^ and PrEP) within their encounters.

We have chosen to approach the relationship between HIV and COVID‐19 through barebacking for several reasons. First, abundant research about barebacking, both from queer and public health approaches, already discusses this practice in terms of risk of HIV, lack thereof, or other affects related to the virus. Second, because of this, barebacking can open necessary discussions about how people make sense of and negotiate HIV. Barebackers may provide first‐hand explorations of the delicate and complex processes of negotiating risk, desire and community in everyday contexts. Finally, we argue that the relationship between barebacking and the risk of HIV (or lack of risk) triggers powerful affective similarities to the COVID‐19 pandemic. Thus, we ask: how do barebackers engage with their experiences of the AIDS crisis and HIV to make sense of COVID‐19? Second, how do they negotiate sexual practices and prevention in a time of COVID‐19 transmission?

## METHODS

The goal of this article was to explore how barebackers engage with COVID‐19 through and alongside their experiences of the AIDS crisis and HIV today and how they negotiate their sexual practices in this context. To do so, we have conducted an online ethnography of a barebacking forum. The process focused on analysing discursive content alongside the dynamics of use, representation and interaction among users and with the forum (see Kozinets, 2015; Hine, 2017 for a summary of this approach). This is a method already used in García‐Iglesias (2021b) as a way of understanding how the content of online discussions is also mediated by the very dynamics, affordances and limitations of these sites (Race, 2015a). The current paper focuses on a well‐known barebacking forum. As of 24 April 2020, the forum featured over 65,000 users whose posts are widely varied in terms of length, quality, topic and the number of replies. While it is unclear where these users are from, the forum's dating section featured mostly locations in the United States, the United Kingdom and Australia. Equally, the cultural and political references in the users’ posts reinforce the idea that the forum is mostly populated by users from the United States and the United Kingdom. Despite these limitations, the forum, with over 70,000 daily active users, remains a key element of barebackers’ sociality online and thus a platform from which to gauge general feelings and discourses within this subculture, even if local and national practices and contexts vary.

We conducted an online ethnography exclusively in the public section of the forum, between 1 July and 9 July 2020. This was a period of wide‐spread crisis: The early summer of 2020 was marked by high infection rates and hospital occupancy, uncertainty about the origins, spread and treatment of the virus and scarcity of personal protective equipment. At the time, vaccines for COVID‐19 seemed still months (if not years) away. We used the search tool provided by the platform to look for posts containing the term ‘COVID’ and ‘corona’. This returned 112 individual posts which were screened. We focused on posts that met established criteria based on significance of risk negotiation and sex, and relevance to the COVID‐19 pandemic. First, the posts should address the interface between barebacking and COVID‐19, as opposed to just providing updates on policy or the epidemic. Second, the posts should develop speculative or creative approaches or feature participants sharing their own experiences or anecdotes of ‘life during COVID‐19’. Third, we were also particularly interested in posts evidencing how users share and negotiate different views and experiences of the virus and the need for prevention measures. This selection process is not dissimilar from identifying relevant interview excerpts (e.g. García‐Iglesias, 2020b; Tillmann, 2015). All relevant posts were downloaded to a shared document. Independently, each author read and identified salient themes in the posts. Authors then jointly discussed the themes, established a set of codes and independently coded and then jointly coded the posts.

The current work builds on existing research on online interactions and sexual practices (e.g. Ferreday, 2009; García‐Iglesias, 2020c). As with all online research, it is particularly difficult to characterise the ‘user behind the screen name’. It is challenging to track users’ posts over time, and it is not possible to individually interview users. This is not unique to this piece, though. Dawson et al. (2005), talking about HIV and online dating advertisements, argue that ‘it may be a leap of faith to equate advertisers’ report of their HIV status [on the sex advertisement site] with their actual serostatus. It is not uncommon to have misrepresentation in Internet profiles' (80). Thus, it is true that there are not feasible ways of determining whether the ‘users behind the posts’ identify as barebackers or whether they engage in barebacking in their offline lives. However, in this article, we use the term *barebackers* because we assume that those users who take the time to create a username and contribute to the site do so because they are interested in or aroused by the discussions and topics therein, even if this is just momentarily. That is, they may not define themselves as barebackers in their daily lives but they are nonetheless participating in the community of the forum while they comment, post or reply. Similar to this dynamic, user profiles provide little information about their owners. This makes it challenging to determine the age of those taking part in these discussions (and, in turn, what their experiences of the AIDS crisis, HIV prevention, etc. maybe). For this article, we rely on their own descriptions of how they lived through the AIDS crisis or came into adulthood in its aftermath. Furthermore, neither the posts selected here nor the users who wrote them should be taken as spokespeople for the entirety barebacking. Our goal was to explore how barebackers negotiated their desires for barebacking and risk during COVID‐19.

Similarly, the fact that the forum is largely anonymous (i.e. usernames do not come with demographic information) means that it is impossible to obtain a representative sample. However, rather than viewing this as an obstacle, we suggest that this perceived anonymity allows users to engage in speculation and discussions that they would not partake in non‐anonymous spaces. Thus, we argue that exploring this barebacking forum is particularly interesting in how it reveals users' speculative processes of negotiating COVID‐19 and HIV from barebackers' perspective.

## FINDINGS AND DISCUSSION

### From AIDS to COVID‐19: negotiating viruses and sex

Despite Florêncio's (2020) argument that barebackers may have decoupled their sex from the spectre of AIDS or HIV risk thanks to new prevention and treatment methods, AIDS and HIV remain central to many discussions in the forum under analysis. Some users discuss how to prevent HIV infection by means of serosorting (i.e. selecting partners based on perceived HIV status), seropositioning (i.e. choosing sexual positions—e.g. ‘top’ or ‘bottom’—based on perceived risk of HIV transmission) or other risk‐reduction techniques. Others focus on the reduced risk of HIV thanks to PrEP, PEP and undetectability. In one of the largest threads dealing with both viruses, a senior user shares screenshots from a Twitter account of a man who gloats about having had sex without condoms (barebacking) with multiple partners while in isolation for COVID‐19. The Twitter user later reveals that he has been hospitalised for COVID‐19 complications. The forum user titles his thread ‘Don't be a Covidiot’ (a portmanteau between COVID and idiot) and comments:

“So the guy is under ‘lockdown’ (a stay‐at‐home order), and he's still hooking up – and proud enough of it to post it on Twitter. […] 4 days after the first hookup, he posts a video of another hookup.… […] 3 days after the 2nd hookup he posts pics and videos from yet another hookup… […] 3 hookups later he's in the hospital with COVID‐19.”

The title and comments by the user anticipate the negative responses that the post receives, with many other users shaming and condemning what they see as recklessness. One user replies:

“What an idiot. I survived the 80's crisis with HIV when so many of my friends died. 13 funerals in 1 year and I'm not looking for a repeat of that time. I don't know how old this asshole is and maybe he didn't live through that time but that's no excuse for not being aware and putting not only himself at risk but others. And I want to point out his playmates are just as irresponsible as he is.”

Even if we do not know his age, this user demonstrates having lived through the AIDS crisis and employs his seeming experiences with health and illness during the height of the AIDS crisis as a tool to appraise and respond to someone else's sexual practices during COVID‐19. Deploying an experiential divide between those who lived through the AIDS crisis and those who did not, he suggests that both groups should be held equally responsible for their actions. He laments the risk to which the Twitter user subjects others and calls both the individual and his ‘fuck buddies’ ‘irresponsible’. These feelings are shared by many others on the forum who reply in similar terms and reinforce the idea of ‘irresponsibility’, returning to their experiences of the AIDS crisis as a comparator:

“I see a lot of irresponsible folks here

Stupid is as stupid does. I made it through the whole AIDs crisis. Not necessarily by ‘safe sex’ but by thought out sex with the least risks, and it worked.”

As much as many users draw on their experience of the AIDS crisis as a comparator, they also refuse to establish too close a similarity between both experiences:

“People, seriously, be responsible! Hospitals are over capacity. People are dying. There's no cure. There isn't even a decent treatment. Even in the middle of the AIDS crisis hospitals could manage their core caseloads. This is a whole other order of magnitude above that pandemic.

This [COVID‐19] is different since the virus is spread just by being near each other before any physical contact is made. So my big 'ol ass will be cherry again when this is over and will be available for an Alpha Top to pop!”

These two users share their lived experience of the AIDS crisis but do so to claim that the COVID‐19 pandemic is different in quality (e.g. routes of transmission, treatment and cure) and quantity (e.g. number of cases). In doing so, they emphasise that their past experience of the AIDS crisis renders them as ‘voices of experience’ but, at the same time, argue that the magnitude of the COVID‐19 pandemic is drastically different from the AIDS crisis as they remember it. Even if these are not entirely accurate statements (hospitals, e.g. did refuse treating patients during the AIDS crisis; see, e.g. France, 2017), these feelings are shared widely among the users who contribute to the forum.

However, not all posts in the forum are based on the AIDS crisis. Because of the seeming varied ages and lived experiences of the forum users, conversations move fluidly between talking about COVID‐19 in relation to the height of the AIDS crisis and to the current context of HIV (as a manageable chronic condition preventable through biomedical interventions, such as PrEP, PEP and TasP). The very nature of the platform allows all users to add comments, which generates a complex conversation that may jump from the memories of AIDS to HIV today. Thus, there are also plenty of users who compare COVID‐19 to a more contemporary framework of HIV. One such user narrates a recent encounter he had on Grindr, a popular gay dating and hook‐up app, and explains that the following conversation took place:

“I asked the guy: ‘So…what's your status? Have you been tested?’ ‘Yeah, man – I'm poz but undetectable.’ ‘No! I'm asking if you have coronavirus. Who cares if you're poz right now?’”

In this recalled exchange, the user mobilises language that is frequently associated with HIV today (e.g. ‘status’ and ‘tested’) to, then, almost humorously break our expectations by showing how it is COVID‐19 to which this language refers. In so doing, he shows how, for him, concerns about HIV in 2020 have been displaced from the pre‐sex negotiations. This is further emphasised by the phrase: ‘who cares if you're poz right now?’ The user shows how ‘undetectability’, and the push towards heightened public awareness of the ‘undetectable equals untransmittable’ (U = U) slogan, has rendered the risk of HIV negligible for him. However, this user also exemplifies how concern about COVID‐19 has taken a pre‐eminent place in sexual negotiations. In his anecdote, risk negotiation no longer only concerns HIV but also, and perhaps even more pressingly, COVID‐19.

As explained above, the users of these posts recall both experiences of the AIDS crisis and the current context of HIV as a framework that helps them to interpret the COVID‐19 context while also drawing out differences between the pandemics. Users emphasise that COVID‐19 is ‘something different’ that requires a new set of prevention techniques and approaches. If these users establish HIV and COVID‐19 as different (in relation to their sexual practices), the question turns to how this difference is experienced.

### ‘Being legs up in a bathhouse’: negotiating subcultural practices

Amidst negotiation of the similarities and differences between COVID‐19 and HIV/AIDS pandemics, users' discussions of intervention strategies are entangled within larger frameworks of barebackers' subcultural practices. On the one hand, they engage in conversations about what behaviours are permissible. On the other hand, they consider to what extent these subcultural sexual practices can be continued while maintaining a sense of shared community and interaction. In this way, they provide discourses of preferred or ‘responsible’ behaviour, which might impede COVID‐19 transmission and preserve the possibility(ies) of their sexual subculture. Some users suggest:

“Avoiding big crowds is important – so no busy bars, ride a bike not the subway, do 1‐on‐1 hookups rather than a sex party, and so on. Also, learn to wash your hands frequently after being in public.

It's gonna be a long time (probably years) before we're back to where we were before with big sex parties.”

Users such as these suggest modifying their practices in a way that minimises risk (‘do 1‐on‐1 hookups rather than a sex party’) yet also serve to continue barebacking sexual practices. However, the second user seems to imply, these modified practices may be somewhat unsatisfactory, as he longs for practices, such as sex parties, which are deferred to an uncertain future.

The impact of their ‘responsible’ behaviour is presented in forum discussions of the future of bathhouses. Bathhouses are commercial venues where gay men engage in sexual activity, frequently in groups or in open areas (see Chauncey, 1994, 207–225). Bathhouses play a key role in the barebacking community, not least because they are a key element in its early history and are seen as ‘safe spaces’ for barebackers (Chauncey, 1994; Delany, 1999; Woods & Binson, 2003). To this date, they remain a space where condomless anal intercourse is normalised (Dean, 2009). Bathhouses are a key concern of forum users, who frequently share advice on what venues are better, tips on visiting them or experiences they have had in them.

In the forum, bathhouses remain at the forefront of discussions about the survival of barebackers' subcultural sexual practices, as evidenced by multiple users who open discussions about the future of bathhouses. Some discuss this in terms of the commercial viability of these spaces:

“Not only will bathhouses be strained on their margins by a percentage of their patrons opting to stay away, this will present a golden ‐ if not irresistible ‐ opportunity for moralistic elements to move to have them closed down by authorities as a menace to public health.

I'm sure this will be the final blow to a lot of bathhouses. Many of them barely hanging on the last few years. Can you imagine what their insurance premiums will be like if they can even get liability insurance?”

At the same time, others focus on the survival of the very social communities that existed within these spaces. For example, one of the most popular users (meaning a user who frequently posts and is respected by other users) suggests further ways to modify practices while also commenting on how ‘ideal’ barebacking may not happen until a distant future:

“Even when the stay‐at‐home orders are lifted people will still need to be vigilant to avoid another peak of the pandemic. You'll need to focus on risk reduction – fewer partners, 1‐on‐1s, maybe sex in parks, maybe gloryholes. But being legs up in a bathhouse taking all loads isn't going to happen again until there's at least a decent treatment. If for no other reason, that sensible gay guys are gonna stay away.”

This user projects an ideal situation (‘being legs up in a bathhouses’) which is deferred ‘until there is at least a decent treatment’. Within this uncertainty, the user suggests that ‘sensible gay guys’ will stay away to observe risk‐reduction practices. It is worth noting how the use of the phrase ‘risk reduction’ in this post confirms how discourses, experiences and memories of HIV and AIDS have influenced these men's understanding of risk and COVID‐19.

Neither the calls for responsibility nor the risk‐reduction suggestions are particularly different from those enacted by mainstream media. As one of the users above mentioned, ‘avoiding big crowds is important – so no busy bars, ride a bike not the subway, do 1‐on‐1 hookups rather than a sex party.’ Thus, the discussion of barebacking is linked to that of other activities, such as exercising, bars and riding the subway. This connection between bathhouses, barebackers and larger, more mainstream, public health discourses is at the heart of the forum. For example, one user comments in the thread about bathhouses:

“This is the first time in history where to save humanity we just need to stay at home.”

Through this post, the user incorporates a sense of a shared responsibility to ‘just stay home.’ Though the assertion ‘just stay home’ is not, in fact, specific to the COVID‐19 pandemic—the idea stemming from the concept of *quarantine* which was first notably deployed during a 17th‐century bubonic plague outbreak (Snowden, 2019, 30)—it does provide a telling link to mainstream perceptions of disease intervention: particularly, the desire to interrupt transmission through the sequestration of infected bodies. In highlighting that the responsible thing is to ‘just’ stay at home, this view colludes with nationalistic rhetorics popularised through social media, for instance, the Italian message: ‘Ricordiamoci che ai nostri nonni fu ordinato di andare in guerra, a noi stanno chiedendo di stare sul divano’ (‘Let us remember that our grandparents were ordered to go to war, we are being asked to stay on the sofa’). Thus, forum discussions sometimes transition from discourses specific to barebackers to those about the society at large.

Barebackers themselves, a group that has historically been framed as hedonistic for their search of pleasure and attitude to HIV (even today, when barebacking can be practised without risk of HIV thanks to new prophylactics), discuss individual and social responsibility both at the subcultural and societal level. In encouraging others to maintain socially distancing practices, they complicate perceptions that barebackers are simply reckless. Rather, these users actively negotiate the terms and conditions that will enable the maintenance of their sexual cultures and well‐being within and along their larger societies.

As shown in the excerpts above, forum discussions fluidly move between users who recall vivid memories from the AIDS crisis and those who frame their views in more contemporary times where HIV is a chronic condition. Overall, the users of this barebacking forum evidence a complex negotiation between the desire for sexual pleasure and the responsibility to reduce risks and protect others. This is revealed in many posts:

“The question is for me: will anyone avoid random sex especially saunas, cruising bars, boom stores, etc. even 1:1 hook ups?

Stay at home, if you must, only have 1‐2‐1 sex and avoid groups and saunas at all costs. Maybe try getting a couple regulars, as opposed to randoms.

I guess handcuffs would keep me from touching my face, but as a cocksucker, I'm a little confused as to whether I should wash my hands before or after swallowing a load. The same with rimming – should you wash your hands before licking out a hole?”

These users describe difficulty balancing desires between sexual pleasure and risk reduction, but they do so using language that draws upon complex histories of health intervention and with surprising nuance in their adaptations. The humorous tone of the third post is particularly poignant, pointing out the need for greater clarification of what extent the community should consider particular practices are safe or risky, and whether they can find avenues for engagement by collectively considering modes of risk reduction and personal hygiene.

Given this discussion, COVID‐19 provides an opportunity to think about the diversity of risks associated with their sexual encounters. While HIV remains a significant risk for some barebackers, though not for others, it poses an altogether different risk to COVID‐19. Users consider the body in their discussion of washing hands and using ‘barrier’ methods (e.g. condoms and glory holes), which demands attention to not simply the transmission of sexual fluids but all bodily fluids. That explicit negotiation of HIV risk is subsumed within these larger discussions of personal and community safety in times of COVID‐19 is not surprising given the current options for prevention and treatment of it. Rather, this group adds to their repertoire of risk negotiation the various layers of transmission from two viruses which ultimately pose a problem for social cohesion and subcultural practice.

The users of this forum evidence a careful and complex negotiation of pleasure and prevention, desire and responsibility in the face of COVID‐19. The new reality of COVID‐19 forces these men to juxtapose pleasure and prevention in their posts, with the outcome of discussing multiple modes of intervention and prevention under the banner of ‘responsibility’. As such, these barebackers are not simply focused on a dichotomy between pleasure and prevention. Rather, in processes the multiplicity of risk and infection, they articulate pleasure *and* prevention as the praxis that allows them to build dynamic bareback communities (Heaphy, 2018).

## CONCLUSION

COVID‐19 has caused changes on a global scale, which is already impacting perceptions of sex, relationships and community politics. Sexual subcultures and practice are changing and will continue to change under the uncertain conditions of the ‘new normality’ of COVID‐19. As forum users mentioned, many bathhouses and gay venues may not survive the economic impact of the pandemic. Similarly, the panorama of prevention and treatment may drastically change how some countries structure their public health measures, for example using social distance as a chance to ‘break the chain’ of new infections of HIV (56 Dean Street, 2020). Others will take the opportunity to further criminalise disease transmission (HIV Justice Network, 2020). While COVID‐19 is not a sexually transmitted infection, the close physical intimacy that barebackers desire may be difficult under new paradigms of intimacy and sexuality. We suggest that barebackers are adapting to the changing context and developing subcultural strategies to sustain their sexual practices, desires and prevent COVID‐19.

This ‘new’ normal will also necessarily imply rethinking and reconsidering what the ‘old’ normal was like. As Twitter user @Chompy__ has commented: ‘As I ventured off in public without a mask or gloves, I couldn't help but wonder… was I socially barebacking?’ (Chompy, 2020). Indeed, the potential for the ‘barebacker’ to take on new meanings has expanded dramatically during COVID‐19 pandemic. In this article, we have engaged in one such expansion: We have explored how barebackers deploy their experiences and memories of HIV and AIDS, both past and present, to make sense of the COVID‐19 pandemic and to rethink prevention strategies in order to maintain their subcultural practices. We have also explored how barebackers combine a simultaneous desire for pleasure and for prevention under COVID‐19. The ways in which these men negotiate pleasure and prevention challenge previous characterisations of these groups as intrinsically hedonistic.

At the same time, while this article has focused on barebackers, the discussions of pleasure and risk, desire and prevention, are not unique to this practice. In fact, early ongoing research suggests that a desire for physical and sexual intimacy may be a key motivator for gay men breaking social distancing rules during the UK lockdown period; as much as a quarter of gay men in the United Kingdom might have had casual sex during the lockdown (Hyndman et al., 2021). While it would be easy to assume that these men flouted the need for prevention in their search for pleasure, the barebackers in this article evidence just how complex, ambivalent and complementary both activities are desire and prevention, pleasure and responsibility.

As we move forward to think about what sexuality, sexual health and prevention will look like in the months and years to come, we ought to reflect once more on the relationship between pleasure, intimacy and desire in a pandemic. As Mark J. Blechner writes: ‘Risk of HIV‐infection is serious. But the risk of loss of pleasure and intimacy is also serious’ (Blechner, 2002, 30). The users of this forum, as barebackers, have a long experience of negotiating this balance between the risk of HIV and the activities that bring them pleasure. They now use that experience to negotiate a new viral risk, COVID‐19.

It is not enough to suggest that risk analysis will impede perceived risky behaviour; indeed, various sexual practices thrive on the idea of risk. Therefore, effective intervention and prevention require a level of nuance that is attentive to the multiple and contradictory motivations of sex and sexuality, bringing to the fore the importance of social connection, community interaction, pleasure and desire amidst networks of viral transmission. To ignore, these elements of human sexuality would risk glossing over subcultural practices engaging in unique forms of intervention and prevention. As evidenced here, barebackers in the forum find new ways to engage in sexual practices through their comparisons of HIV and COVID‐19, their discourses of responsibility and their concerns about the maintenance and cohesion of socio‐sexual practices and spaces for the future.

## AUTHOR CONTRIBUTIONS

**Jaime Garcia‐Iglesias:** Conceptualization (equal); Data curation (equal); Formal analysis (equal); Investigation (equal); Methodology (equal); Project administration (equal); Resources (equal); Software (equal); Supervision (equal); Validation (equal); Visualization (equal); Writing‐original draft (equal); Writing‐review & editing (equal). **Chas Ledin:** Conceptualization (equal); Data curation (equal); Formal analysis (equal); Investigation (equal); Methodology (equal); Project administration (equal); Resources (equal); Software (equal); Supervision (equal); Validation (equal); Visualization (equal); Writing‐original draft (equal); Writing‐review & editing (equal).

## ENDNOTES

^1^
This article will describe risk using common parlance, for example COVID‐19 risk. The virus (SARS‐CoV‐2) and disease (COVID‐19) are discrete concepts which, together, describe the etiology of this pathology. As the World Health Organization (2020) suggests, viruses receive their names based on genetic structures, whereas diseases receive their names based on myriad factors related to ‘disease prevention, spread, transmissibility, severity and treatment’. We critically engage with the myriad social, cultural and biological factors implicated in disease transmission and thus refer to this phenomenon as ‘COVID‐19’.

^2^
We include under these terms a wide array of possible memories and experiences of HIV and AIDS, including those who lived through the AIDS crisis, those who have only known HIV as a chronic manageable condition, those who have contracted the virus, etc.

^3^
For the purposes of this article, we define the early AIDS crisis as the period between 1981 and 1996, during which high rates of HIV incidence, deaths and cultural movements (e.g. ACT UP New York) occurred. As Cheng et al. (2020) suggest, AIDS crises are diverse and manifest unevenly across time, history and geolocation, and, in many cases, crises are ongoing.

^4^
We establish the separation between the AIDS crisis (see previous footnote) and the current context of HIV, which starts after the development of ART in 1996 and continues, today, with the availability of effective treatment and prevention mechanism. We remain aware, however, that this separation ignores the fact that, for many communities, such as Black communities or trans people, HIV remains a crisis to this day (see Cheng et al., 2020).

^5^
Antiretrovirals (commonly called ARVs, ART or HAART) are a class of medications, which are used in combination (aka regimes) to treat HIV infection.

^6^
Pre‐exposure prophylaxis (commonly called PrEP) is a combination of HIV medicines which HIV‐negative individuals take before potential exposure to prevent the transmission of HIV.

^7^
‘Undetectable equals Untransmittable’ (U = U) is a public health slogan that describes medical consensus that people living with HIV on effective treatment and with undetectable viral loads cannot pass on the virus to HIV‐negative partners.

^8^
Post‐exposure prophylaxis (commonly called PEP) is a combination of HIV medicines which individuals who have been exposed to HIV can take within 72 h of infection. PEP is not a cure. Regimes must be taken for 28 days and do not guarantee successful prevention of HIV infection.
